# MiR-99a-5p up-regulates LDLR and functionally enhances LDL-C uptake via suppressing PCSK9 expression in human hepatocytes

**DOI:** 10.3389/fgene.2024.1469094

**Published:** 2024-11-19

**Authors:** Xuemei Chen, Ying Liu, Qiujing Zhou, Chenxi Zhang, Wei Wang, Menglong Xu, Yaqiang Zhao, Wenfeng Zhao, Dian Gu, Shuhua Tan

**Affiliations:** Department of Cell and Molecular Biology, School of Life Science and Technology, State Key Laboratory of Natural Medicines, Jiangsu Key Laboratory of Druggability of Biopharmaceuticals, China Pharmaceutical University, Nanjing, Jiangsu, China

**Keywords:** microRNA, pcsk9, LDLR, LDL-C, hypercholesterolemia, atherosclerosis

## Abstract

**Background:**

MicroRNAs (miRs/miRNAs) play pivotal roles in modulating cholesterol homeostasis. Proprotein convertase subtilisin/kexin type 9 (PCSK9) binds to low-density lipoprotein receptor (LDLR) at the surface of hepatocytes and accelerates its degradation in lysosomes, thereby impairing the clearance of circulating low-density lipoprotein cholesterol (LDL-C) from plasma. Thus, suppressing PCSK9 expression level has become an effective approach for treating hypercholesterolemia. Here, we sought to identify novel miRNAs that inhibit PCSK9 expression.

**Methods:**

By *in silico* analyses, miR-99a-5p was predicted to bind to human *PCSK9* mRNA. Following transfection of miR-99a-5p or anti-miR-99a-5p in human and mouse hepatocytes, qRT-PCR, Western blot, immunofluorescence, ELISA, flow cytometry, LDL-C uptake, and cellular cholesterol measurement were performed.

**Results:**

miR-99a-5p overexpression potently inhibited PCSK9 expression, thereby up-regulating LDLR, functionally enhancing LDL-C uptake and increasing intracellular cholesterol levels in human, but not in mouse, cells. Conversely, anti-miR-99a-5p upregulates PCSK9, leading to a reduction in LDLR, attenuation of LDL-C uptake, and a decrease in the intracellular cholesterol levels of human hepatocytes. Furthermore, miR-99a-5p was shown to bind to the predicted target site “UACGGGU” in the 3′-UTR of human *PCSK9* mRNA via a luciferase reporter assay in combination with site-directed mutagenesis.

**Conclusion:**

MiR-99a-5p potently downregulates the expression of PCSK9 by directly interacting with a target site in the human *PCSK9* 3′-UTR, thereby up-regulating LDLR and functionally enhancing LDL-C uptake in human hepatocytes. MiR-99a-5p could serve as an inhibitor of PCSK9 for treating hypercholesterolemia to inhibit atherosclerosis.

## 1 Introduction

Hypercholesterolemia, especially elevated plasma low-density lipoprotein cholesterol (LDL-C) levels, is known to be one of the pivotal risk factors for atherosclerosis (AS) and atherosclerotic cardiovascular disease (ASCVD) (Ference et al., 2017). Circulating LDL-C is cleared primarily by hepatic low-density lipoprotein receptor (LDLR)-mediated endocytosis (Garcia et al., 2001). Once plasma LDL-C binds to hepatocyte surface LDLR, the LDL-C/LDLR complex is internalized by clathrin-coated pits and transported to endosomes, where LDL-C dissociates from the complex and is degraded in lysosomes and LDLR is recycled back to the surface of hepatocytes (Luo et al., 2020). Thus, increasing LDLR levels to increase LDL-C uptake may be an effective treatment for hypercholesterolemia and ASCVD.

Proprotein convertase subtilisin/kexin type 9 (PCSK9) is a secreted serine protease containing 692 amino acid residues that comprises a signal peptide, a pre-structural domain, a catalytic structural domain, and a C-terminal structural domain and is synthesized mainly in hepatocytes (Seidah et al., 2003). PCSK9 is initially synthesized as an inactive zymogen, followed by autocatalytic cleavage in the endoplasmic reticulum (ER) at the VFAQ_152_↓SIP site, which is required for its trafficking from the ER to the secretory pathway (Benjannet et al., 2004). After cleavage, mature PCSK9 remains noncovalently attached to the prodomain to prevent other substrates from interacting with it; thus, proteolytic activity of PCSK9 is avoided through the secretion pathway. By binding the extracellular epidermal growth factor-like repeat A (EGF-A) structural domain of LDLR, PCSK9 promotes the degradation of LDLR in lysosomes, thus preventing its recycling and reducing LDL-C clearance (Bergeron et al., 2015). Consequently, suppressing PCSK9 expression to reduce PCSK9-mediated degradation of LDLR has become a promising therapeutic way to reduce circulating LDL-C levels (Norata et al., 2014; Della Badia et al., 2016).

MicroRNAs (miRs/miRNAs) are small (∼22 nt), highly conserved, endogenous, single-stranded, non-coding RNAs that regulate genes by binding to the complementary sequences of their target mRNAs to promote their degradation and/or translational inhibition (Bartel, 2009). Studies have shown that miRNAs are related to many pathological conditions ranging from metabolic disease to cancer and can potentially be exploited as promising therapeutic targets or agents (Loyer et al., 2015; Krutzfeldt, 2016; Regazzi, 2018; Wang et al., 2021). To date, numerous miRNAs have been confirmed to be crucial posttranscriptional regulators of genes involved in lipid metabolism (Aryal et al., 2017). Among them, miR-33 suppresses expression of ATP-binding cassette transporter protein A1 (ABCA1), resulting in decreased plasma levels of high-density lipoprotein (HDL) (Rayner et al., 2010). On the other hand, miR-33 antagonism not only increases hepatic ABCA1 expression but also increases expression of its target genes that participate in fatty acid oxidation and lowers the expression of genes involved in fatty acid synthesis, thus increasing HDL and decreasing VLDL triglyceride levels (Rayner et al., 2011). In addition, miR-148a, miR-128-1, miR-301b, miR-130b (Wagschal et al., 2015), miR-27a (Alvarez et al., 2015), miR-27b (Goedeke et al., 2015), miR-185 (Jiang et al., 2015), and miR-140-5p (Xu et al., 2020) have been shown to modulate the expression of LDLR. MiR-224 (Bai et al., 2017; Naeli et al., 2017; Salerno et al., 2020), miR-222, miR-191 (Naeli et al., 2017), miR-520d (Salerno et al., 2020), miR-337-3p (Xu X. et al., 2021), miR-483 (Dong et al., 2020) and miR-552-3p (Ma et al., 2021) have been shown to regulate PCSK9 expression.

In this work, we sought to identify novel miRNAs that inhibit PCSK9 expression in human hepatocytes. As a result, miR-99a-5p targets the 3′-UTR of human *PCSK9* mRNA and suppresses its expression, thereby increasing LDLR levels and functionally enhancing LDL-C uptake in human hepatocytes. These data suggest that miR-99a-5p may be a potential therapeutic agent to ameliorate hypercholesterolemia and inhibit atherosclerosis.

## 2 Materials and methods

### 2.1 Reagents and media

DMEM (Cat# 12800017), MEM (Cat# 41500034), Opti-MEM (Cat# 31985070) and fetal bovine serum (FBS, Cat# 10099141) were purchased from Gibco (Grand Island, NY, United States). Lipofectamine 3000 (Cat# L3000015) was obtained from Invitrogen (Carlsbad, CA, United States). Rabbit monoclonal anti-β-actin antibody (Cat# 4970) was purchased from Cell Signaling Technology (Danvers, MA, United States). Rabbit monoclonal anti-LDLR antibody (Cat# ab52818), rabbit polyclonal anti-PCSK9 antibody (Cat# ab95478), monoclonal anti-PCSK9 antibody (Cat# ab181142) were ordered from Abcam (Cambridge, United Kingdom). Alexa Fluor^®^ 488-conjugated goat anti-rabbit IgG (H + L) (Cat# FMS-RBaf48801) and HRP-conjugated goat anti-rabbit IgG (H + L) (Cat# FMS-RB01) were bought from FcMACS (Nanjing, China). RIPA lysis buffer (Cat# R0020) and phenylmethyl sulfonyl fluoride (PMSF, Cat# P0100) were purchased from Solarbio (Beijing, China). RNAiso Plus (Cat# 9108) and PrimeScript™ RT reagent Kit with gDNA Eraser (Cat# RR047A) as well as TB Green^®^ *Premix Ex Taq*™ II (Tli RNaseH Plus) (Cat# RR820A) were obtained from Takara (Dalian, China). Human PCSK9 ELISA Kit (Cat# EK1124-96) was obtained from Multi Sciences (Hangzhou, China). MiRNA mimics and inhibitors were from GenePharma (Shanghai, China). The pmirGLO Dual-Luciferase miRNA Target Expression Vector (Cat# E1330) and Dual-Luciferase^®^ Reporter Assay System (Cat# E1910) were purchased from Promega (Madison, WI, United States). Dzup Genomic DNA Isolation Reagent (Cat# B518201) was bought from Sangon Biotech (Shanghai, China). Oxidized low-density lipoprotein (ox-LDL, Cat# YB-002) and 1, 1′-dioctadecyl-3, 3, 3′, 3′-tetramethyl-indocarbocyanine perchlorate labeled LDL (DiI-LDL, Cat# YB-0011) were obtained from Yiyuan Biotechnologies (Guangzhou, China).

### 2.2 Bioinformatics analysis

The miRNAs that could target the 3′-UTR of human *PCSK9* mRNA (RefSeq ID: NM_174,936; Ensembl ID: ENSG00000169174/ENST00000302118.5) were identified via various online target prediction algorithms, including miRanda (http://www.microrna.org/microrna/home.do) (Betel et al., 2010), TargetScan 7.2 (http://www.targetscan.org/vert_72/) (Agarwal et al., 2015), miRDB (http://www.mirdb.org/miRDB/) (Liu and Wang, 2019). As a result, 19 miRNAs were identified by miRanda analysis with good mirSVR scores (≤−0.1), and 106 miRNAs were obtained based on TargetScan 7.2 analysis with the context++ score percentile ≥90, and 49 miRNAs were selected according to miRDB analysis with target score ≥50. Then, 10 miRNAs were selected with good prediction scores via venn diagram. In addition, miRNAs were identified via the miRBase database (http://www.mirbase.org/) (Kozomara et al., 2019).

### 2.3 Cell cultures

HepG2 (human hepatoma cells), LO2 (human hepatic cells), and Hepa1-6 (mouse hepatoma cells) were acquired from National Infrastructure of Cell Line Resource (Beijing, China). The cells were cultured in DMEM (LO2 and Hepa1-6 cells) or MEM (HepG2 cells) supplemented with penicillin (100 U/mL), streptomycin (100 μg/mL) and 10% FBS at 37°C in 5% CO_2_. After transfection for 48 h, the culture medium was replaced with Opti-MEM for quantification of secreted PCSK9 (PCSK9-S), LDLR, and DiI-LDL uptake.

### 2.4 Transfections of miRNA mimics/inhibitors

HepG2 or LO2 cells were inoculated at 5 × 10^5^ cells per well in 6-well plates and transfected with either 50 nM control miRNA mimic (Con miR, sense 5′-UUC​UCC​GAA​CGU​GUC​ACG​UTT-3′) (Cat# B04002), 50 nM miR-99a-5p mimic (miR-99a-5p, sense 5′-AAC​CCG​UAG​AUC​CGA​UCU​UGU​G-3′) (Cat# B02001), 50 nM control miRNA inhibitor (Con Inh, 5′-CAG​UAC​UUU​UGU​GUA​GUA​CAA-3′) (Cat# B04003), or 50 nM miR-99a-5p inhibitor (anti-miR-99a-5p, 5′-CAC​AAG​AUC​GGA​UCU​ACG​GGU​U-3′) (Cat# B03001) utilizing lipofectamine 3000 according to the manufacturer’s instructions. Forty-8 h later, the mRNA levels of *PCSK9* were quantified via qRT-PCR. The cells were cultured in Opti-MEM for another 24 h at 37°C in 5% CO_2_. The protein levels of intracellular PCSK9 and PCSK9-S were detected by Western blot, immunofluorescence and ELISA, respectively. LDLR protein and LDL-C uptake were assessed by Western blot, flow cytometry, immunofluorescence and DiI-LDL staining. For the dose-response studies, following the transfection of HepG2 cells with 50 nM Con miR or various concentrations of miR-99a-5p (25, 50, 100 nM), or with 50 nM Con Inh or increasing concentrations of anti-miR-99a-5p (25, 50, 100 nM) as described above, the cells and the culture supernatant were collected for qRT-PCR, Western blot and ELISA, respectively.

### 2.5 qRT-PCR analysis

Total RNA was isolated from HepG2 or LO2 cells by using RNAiso Plus reagent and quantified by measuring A_260 nm_ with NanoDrop 2000 (Thermo Fisher Scientific). Reverse transcription of 1 μg of total RNA to cDNA was performed using the PrimeScript™ RT kit with gDNA eraser. Then, qRT-PCR analyses were performed in triplicate via TB Green^®^ *Premix Ex Taq*™ II (Tli RNaseH Plus) in an Mx3000P qRT-PCR instrument (Agilent Technologies, Santa Clara, CA, United States). The primers used are listed in Supplementary Table S1. The relative mRNA expression levels were calculated via the 2^−ΔΔCt^ method (Livak and Schmittgen, 2001) and normalized to those of the housekeeping gene *β-actin*.

### 2.6 Western blot analysis

Protein levels were measured via Western blot analysis as previously described (Xu M. et al., 2021). The cells were lysed in RIPA lysis buffer supplemented with 1 mM PMSF for 30 min. After centrifugation at 12,000 rpm for 15 min at 4°C, the supernatant was harvested. A bicinchoninic acid (BCA) protein assay kit (Biomiga, San Diego, CA, United States) was used to quantify the total protein concentration. 40 μg total protein isolated via 10% SDS-PAGE was transferred to a 0.2 μm polyvinylidene fluoride (PVDF) membrane (Merck Millipore, Darmstadt, Germany). After blocking with 5% (wt/vol) nonfat milk/TBST for 1 h at room temperature, the membranes were incubated with primary antibodies against β-actin (Cat# 4970, 1:1,000), PCSK9 (Cat# ab181142, 1:2000) and LDLR (Cat# ab52818, 1:1,000) at 4°C overnight, and then incubated with HRP-conjugated goat anti-rabbit IgG (H + L) (Cat# FMS-RB01, 1:5,000) for 1 h at room temperature. After washing with TBST, the membranes were exposed to ECL (Thermo Scientific, Massachusetts, United States), and the protein bands were quantified using ImageJ software.

### 2.7 Immunofluorescence analysis

Immunofluorescence was conducted to detect PCSK9 and cell-surface LDLR protein levels as previously described (Xu et al., 2020; Xu M. et al., 2021). Briefly, the cells were washed and subsequently fixed in 4% (wt/vol) paraformaldehyde/PBS for 30 min. For detection of PCSK9 expression, the cells were incubated with permeabilization solution (0.1% (vol/vol) Triton X-100/PBS) for 15 min at room temperature and washed with PBS. The cells were then blocked with 10% (vol/vol) goat serum/PBST for 1 h at room temperature and incubated with a rabbit polyclonal anti-PCSK9 antibody (Cat# ab95478, 1:50) and a rabbit monoclonal anti-LDLR antibody (Cat# ab52818, 1:100) overnight at 4°C. Thereafter, the cells were washed, and incubated with Alexa Fluor^®^ 488-conjugated goat anti-rabbit IgG (H + L) (Cat# FMS-RBaf48801, 1:300) for 1 h at room temperature, and then counterstained with DAPI (KeyGEN BioTECH, Nanjing, China) for 10 min at room temperature. Subsequently, fluorescence images were acquired via a confocal laser scanning microscope (CLSM, LSM700, Zeiss, Germany).

### 2.8 ELISA

Secreted PCSK9 in the culture supernatant was detected via ELISA via a human PCSK9 ELISA kit (Cat# EK1124-96, Multi Sciences, Hangzhou, China) following the manufacturer instructions. The optical density at 450 nm and 570 nm was determined by using a multimode microplate reader (Thermo Scientific, Massachusetts, United States).

### 2.9 Luciferase reporter assay

The 3′-UTR of human *PCSK9* gene (1,098 bp) was amplified from genomic DNA of HepG2 cells by PCR and cloned to the pmirGLO Dual-Luciferase miRNA Target Expression Vector between the Xho I and Xba I sites of the 3′-terminal of the firefly luciferase (*luc2*) reporter gene (Figure 4B). The mutation in the miR-99a-5p targeting site within the 3′-UTR of human *PCSK9* was generated by overlap extension PCR utilizing the specific mutagenic primers provided in Supplementary Table S2. HepG2 cells were co-transfected with the indicated 3′-UTR luciferase reporter plasmids and 50 nM Con miR, miR-99a-5p, Con Inh or anti-miR-99a-5p. After transfection for 24 h, the luciferase activities were detected via the Dual-Luciferase^®^ Reporter Assay System.

### 2.10 Flow cytometry analysis

Flow cytometry analysis was conducted to measure cell-surface LDLR protein levels as previously described (Xu et al., 2020; Xu M. et al., 2021). Cells were digested with trypsin and washed with PBS, and subsequently fixed in 4% (wt/vol) paraformaldehyde/PBS for 10 min. After incubation with PBST for 20 min, the cells were blocked with 10% goat serum for 30 min at room temperature and then incubated with anti-LDLR antibody (Cat# ab52818, 1:100) for 30 min at room temperature, and then incubated with Alexa Fluor^®^ 488-conjugated goat anti-rabbit IgG (H + L) (Cat# FMS-RBaf48801, 1:300) for 30 min at room temperature. Detection was conducted on a Guava EasyCyte™ Flow Cytometer (Merck Millipore, Germany). The cell-surface LDLR levels were detected via FlowJo software (FlowJo, Oregon, United States).

### 2.11 LDL-C uptake assay

The LDL-C uptake assay was performed as previously described (Xu et al., 2020; Xu M. et al., 2021). HepG2 or LO2 cells were inoculated at a density of 1 × 10^4^ cells per well in 96-well black plates and transfected with either 50 nM Con miR, miR-99a-5p, Con Inh, or anti-miR-99a-5p for 48 h. Then, the cells were cultured in Opti-MEM for another 24 h. Subsequently, DiI-LDL (20 μg/mL) was incubated at 37°C for 4 h. After washing with PBS, LDL-C uptake was examined on a fluorescence microplate reader (Varioskan flash, Thermo Scientific) at an excitation wavelength of 520 nm and an emission wavelength of 580 nm.

### 2.12 Cellular cholesterol measurements

Cellular cholesterol was measured as previously described (Xu et al., 2020). Briefly, cells were transfected with either 50 nM Con miR, miR-99a-5p, Con Inh, or anti-miR-99a-5p. Forty-8 h later, the cells were incubated with 50 μg/mL ox-LDL for another 24 h. Then, the intracellular cholesterol content was quantified by measuring A_550 nm_ via a cellular cholesterol assay kit (Applygen Technologies, Beijing, China) on a multimode microplate reader (Thermo Scientific).

### 2.13 Statistical analysis

The values are presented as the means ± SEMs. Unpaired two-tailed Student’s *t*-test and one-way analysis of variance (ANOVA) were used for statistical comparisons with GraphPad Prism 8.0.2 software (GraphPad Software, La Jolla, CA, United States). *P* < 0.05 was considered statistically significant.

## 3 Results

### 3.1 PCSK9-targeting miRNAs are predicted using bioinformatics analyses

**FIGURE 1:**
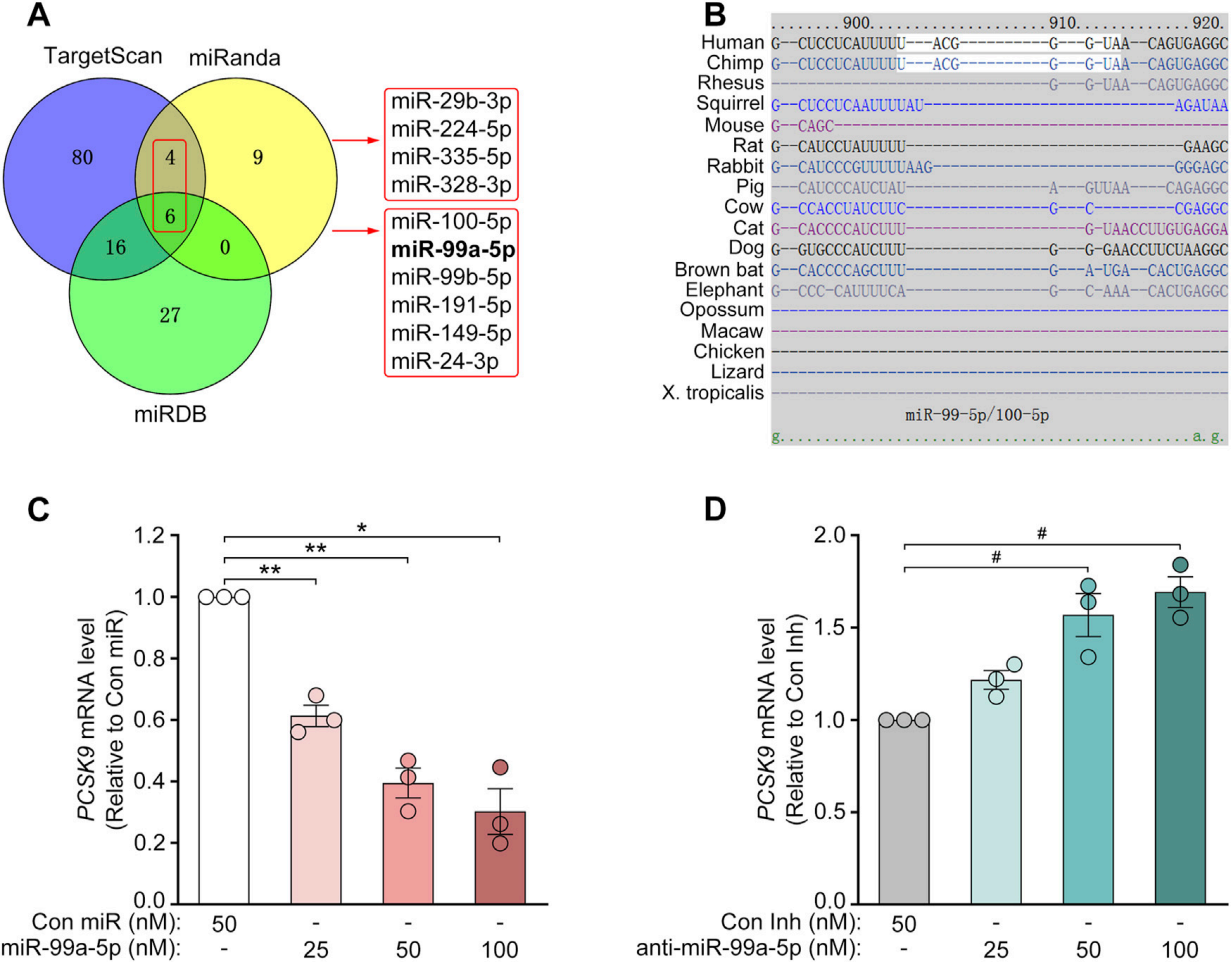
MiR-99a-5p decreases *PCSK9* mRNA levels in human HepG2 cells. **(A)** Venn diagram of the putative human PCSK9-targeting miRNAs predicted by several online target prediction algorithms (miRanda, TargetScan and miRDB). **(B)** The miR-99a-5p seed sequence-interacting site “UACGGGU” in the 3′-UTR of the human *PCSK9* mRNA was predicted via TargetScan 7.2. **(C, D)** *PCSK9* mRNA levels in HepG2 cells determined by qRT-PCR analysis. Data were expressed as the means ± SEM and representative of three independent experiments. ^*^ *p* < 0.05, ^**^ *p* < 0.01 vs. Con miR; ^#^ *p* < 0.05 vs. Con Inh. Significance was performed by one-way ANOVA.

To identify potential PCSK9-targeting miRNAs, we utilized various online target prediction algorithms such as miRanda (Betel et al., 2010), TargetScan (Agarwal et al., 2015) and miRDB (Liu and Wang, 2019), to analyze the 3′-UTR of human *PCSK9* mRNA (RefSeq ID: NM_174,936; Ensembl ID: ENSG00000169174/ENST00000302118.5). First, 19 miRNAs were identified according to miRanda with good mirSVR scores (≤−0.1) (Betel et al., 2010). Then, 10 miRNAs with good prediction scores were selected through comprehensive analyses via TargetScan (Agarwal et al., 2015) and miRDB (Liu and Wang, 2019) (Figure 1A). Among these 10 miRNAs, miR-191-5p, miR-224-5p (Bai et al., 2017; Naeli et al., 2017; Salerno et al., 2020), and miR-24-3p (Shaoliang Zhu, Master’s dissertation of Shanghai University, China) have been previously shown to inhibit PCSK9 expression, whereas miR-328-3p (mirSVR score = −0.1018) has a much poorer prediction score. Thus, we tested the inhibitory effects of the other 6 miRNAs on PCSK9 expression at the protein level via Western blot. As a result, miR-99a-5p suppressed PCSK9 expression most efficiently (Supplementary Table S3; Supplementary Figure S1).

### 3.2 MiR-99a-5p diminishes PCSK9 expression in human hepatocytes, but not in mouse liver cells

Target prediction via TargetScan 7.2 revealed that the 3′-UTR of human *PCSK9* has one putative binding site for miR-99a-5p in humans and chimpanzees but not in mice or rats (Figure 1B). To explore its inhibitory effects on human PCSK9, HepG2 cells were transfected with Con miR or increasing concentrations of miR-99a-5p (25, 50, 100 nM) or with Con Inh or different concentrations of anti-miR-99a-5p (25, 50, 100 nM) for 48 h. Subsequently, qRT-PCR analysis was conducted to measure *PCSK9* mRNA levels. Compared with Con miR, miR-99a-5p downregulated *PCSK9* mRNA expression in a dose-dependent manner (Figure 1C). Conversely, anti-miR-99a-5p induced a dose-dependent increase in *PCSK9* expression at the mRNA level compared with Con Inh in HepG2 cells (Figure 1D). Additionally, *PCSK9* mRNA level was decreased by 56.53% in LO2 cells transfected with 50 nM miR-99a-5p in comparison with Con miR (Figure 3A). Conversely, *PCSK9* mRNA level was up-regulated by 83.23% in LO2 cells transfected with 50 nM anti-miR-99a-5p as compared to Con Inh (Figure 3B). These results verify that miR-99a-5p decreases *PCSK9* mRNA levels in human hepatic cells.

**FIGURE 2:**
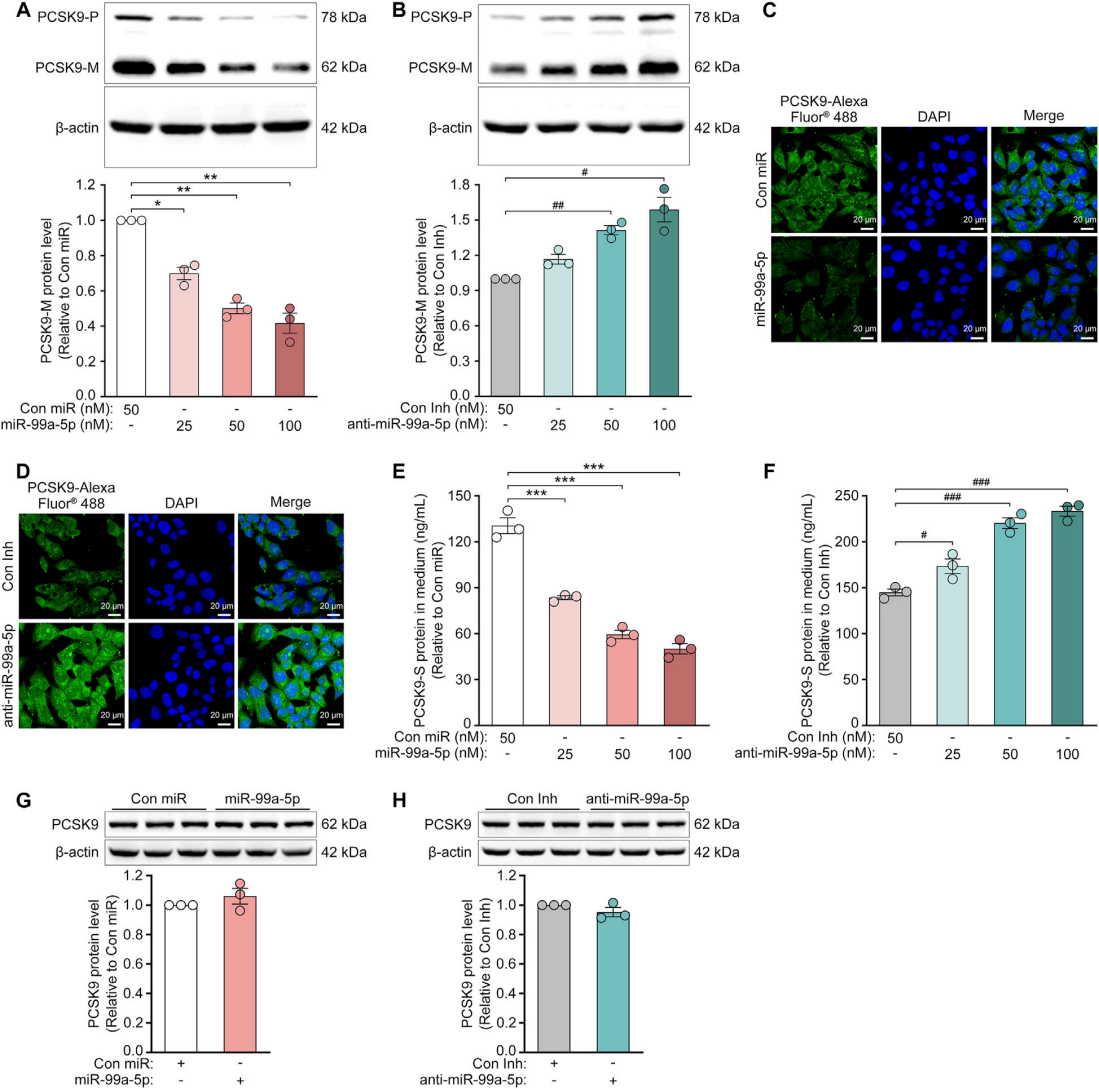
MiR-99a-5p downregulates the protein expression of PCSK9 in human HepG2 cells, but not in mouse Hepa1-6 cells. **(A, B)** Western blot analysis of intracellular precursor PCSK9 (PCSK9-P) and mature PCSK9 (PCSK9-M) in HepG2 cells. **(C, D)** Immunofluorescence analysis of intracellular PCSK9 in HepG2 cells following transfection with either Con miR or miR-99a-5p **(C)**, or with either Con Inh or anti-miR-99a-5p **(D)** at 50 nM. **(E, F)** ELISA analysis of PCSK9-S in the culture supernatant of HepG2 cells. **(G, H)** Western blot analysis of intracellular PCSK9 in mouse Hepa1-6 cells following transfection with either Con miR or miR-99a-5p **(G)**, or with either Con Inh or anti-miR-99a-5p **(H)** at 100 nM. Data were given as the means ± SEM and representative in three independent experiments. ^*^ *p* < 0.05, ^**^ *p* < 0.01, ^***^ *p* < 0.001 vs. Con miR; ^#^ *p* < 0.05, ^##^ *p* < 0.01, ^###^ *p* < 0.001 vs. Con Inh. Significance was analyzed by one-way ANOVA and two-tailed Student’s *t*-test.

**FIGURE 3:**
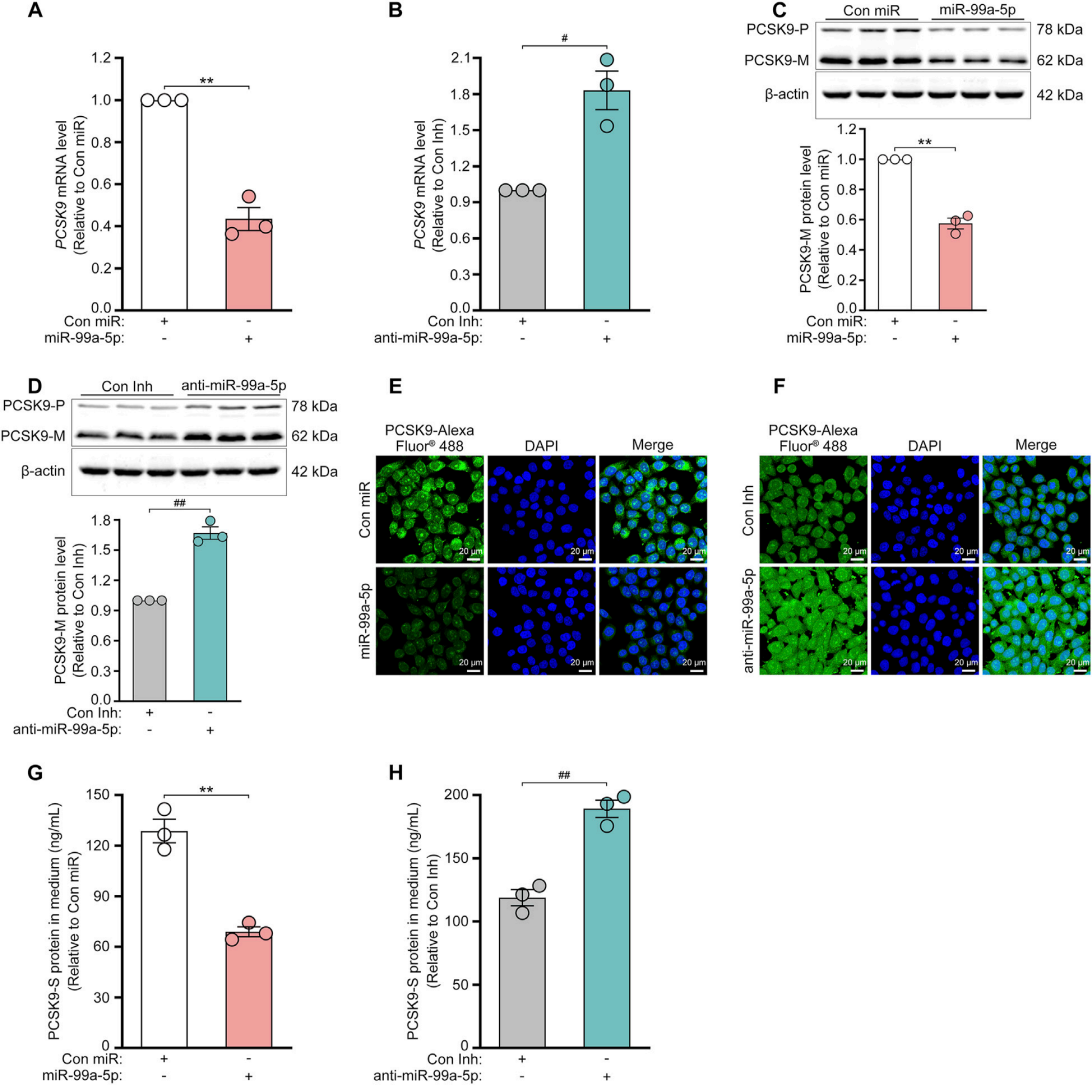
MiR-99a-5p inhibits PCSK9 expression in human LO2 cells. 50 nM Con miR or 50 nM miR-99a-5p, or either 50 nM Con Inh or 50 nM anti-miR-99a-5p were transfected in LO2 cells. **(A, B)** *PCSK9* mRNA levels were quantified by qRT-PCR. Intracellular PCSK9 protein levels were measured by Western blot **(C, D)** and immunofluorescence analysis **(E, F)**, respectively. **(G, H)** The protein concentration of PCSK9-S in the culture supernatant was measured by ELISA. Data were shown as the means ± SEM in three independent experiments. ^**^ *p* < 0.01 vs. Con miR; ^#^ *p* < 0.05, ^##^ *p* < 0.01 vs. Con Inh. Significance was performed by two-tailed Student’s *t*-test.

To further confirm the regulation of PCSK9 by miR-99a-5p, we assessed the protein expression of intracellular PCSK9 by Western blot and immunofluorescence in HepG2 cells. As shown in Figures 2A, C, miR-99a-5p dramatically down-regulated intracellular PCSK9 protein levels in HepG2 cells. In contrast, transfection with anti-miR-99a-5p apparently up-regulated intracellular PCSK9 protein expression (Figures 2B, D). Similar results were also observed in LO2 cells (Figures 3C–F). Additionally, we measured the protein concentration of PCSK9-S in the culture supernatant by ELISA in HepG2 cells transfected with Con miR or various concentrations of miR-99a-5p (25, 50, 100 nM) or with Con Inh or different concentrations of anti-miR-99a-5p (25, 50, 100 nM) for 72 h. As a result, miR-99a-5p decreased PCSK9 secretion from HepG2 cells in a dose-dependent manner (Figure 2E), whereas anti-miR-99a-5p increased secreted PCSK9 levels in a dose-dependent manner (Figure 2F). In addition, PCSK9-S level in the culture supernatant was downregulated by 46.50% in LO2 cells transfected with 50 nM miR-99a-5p compared with Con miR (Figure 3G). In contrast, PCSK9-S level in the culture supernatant was upregulated by 59.26% in LO2 cells transfected with 50 nM anti-miR-99a-5p relative to Con Inh (Figure 3H). These data demonstrate that miR-99a-5p decreases the intracellular and secreted protein levels of PCSK9 in human hepatocytes.

Furthermore, we investigated the role of miR-99a-5p in regulating PCSK9 expression in Hepa1-6 cells, a mouse hepatic cell line, via Western blot. As expected, neither miR-99a-5p nor anti-miR-99a-5p regulated mouse PCSK9 expression at the protein level (Figures 2G, H), which was attributed to the absence of a putative miR-99a-5p binding site in the mouse *PCSK9* 3′-UTR (Figure 1B). These data demonstrate that miR-99a-5p inhibits PCSK9 expression in human hepatocytes, but not in mouse liver cells.

### 3.3 MiR-99a-5p directly targets the human *PCSK9* 3′-UTR

**FIGURE 4:**
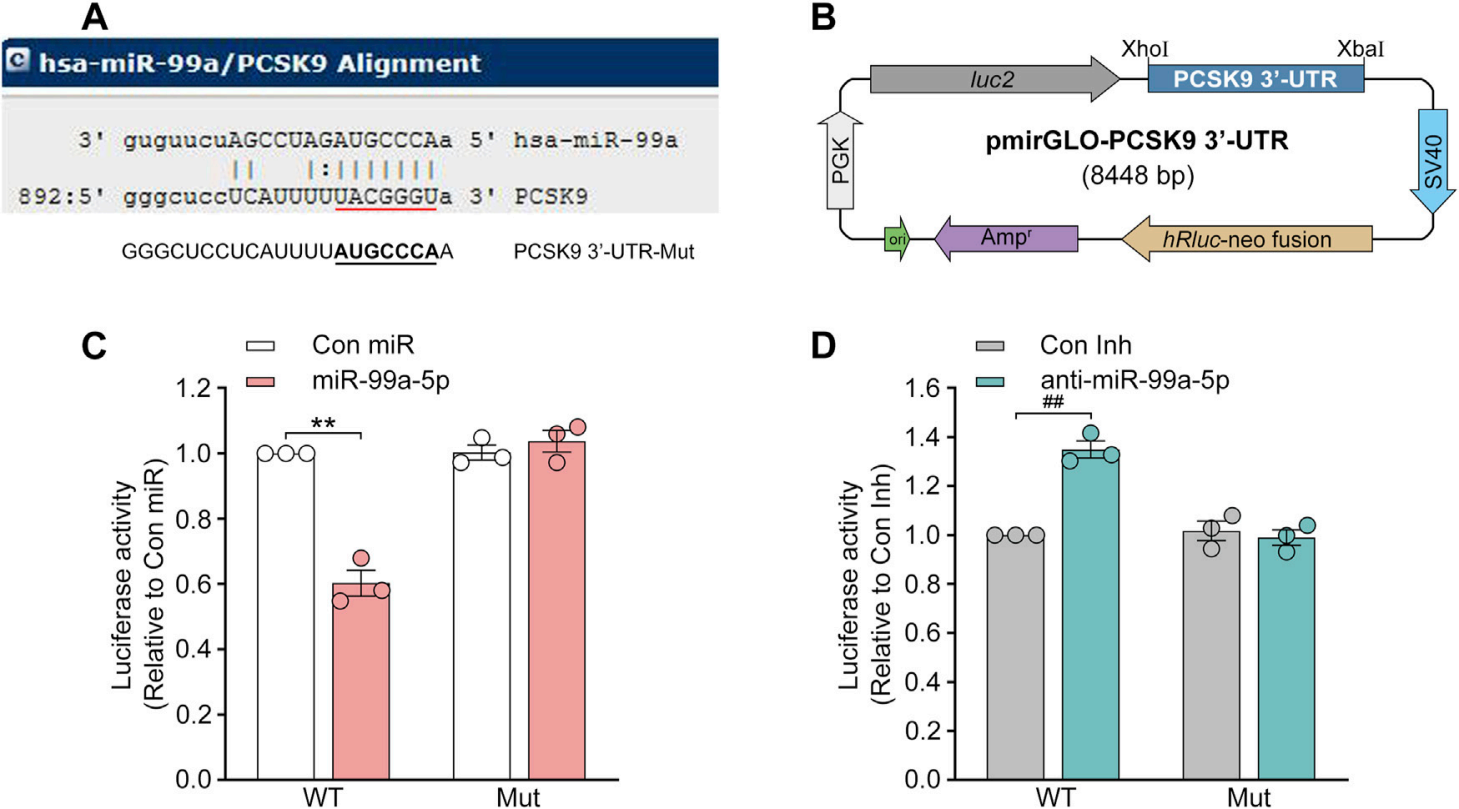
MiR-99a-5p specifically targets human *PCSK9* 3′-UTR to stimulate post-transcriptional degradation. **(A)** The one target site within the human *PCSK9* 3′-UTR predicted via miRanda is indicated in red, whereas the putative binding site was mutated to its complementary sequence underlined in black. **(B)** The 3′-UTR of human *PCSK9* was amplified and cloned at the 3′-end of the *luc2* reporter gene in the pmirGLO vector. **(C, D)** Luciferase activity was detected in HepG2 cells transfected with luciferase reporter plasmids containing WT or mutated human *PCSK9* mRNA 3′-UTR and 50 nM Con miR, or miR-99a-5p **(C)**, or Con Inh, or anti-miR-99a-5p **(D)**. PGK, phosphoglycerate kinase; Mut, mutant. Data were given as the means ± SEM and representative in three independent experiments. ^**^ *p* < 0.01 vs. Con miR; ^##^ *p* < 0.01 vs. Con Inh. Significance was analyzed by two-way ANOVA.

Since miRNAs generally interact with the 3′-UTR of mRNA targets to induce posttranscriptional repression (Izaurralde, 2015; Bartel, 2018), we sought to verify whether miR-99a-5p directly binds to the 3′-UTR of human *PCSK9*. According to the miRanda prediction, the human *PCSK9* 3′-UTR contains a target site for the miR-99a-5p seed sequence (Figure 4A). Thus, the luciferase reporter plasmids were constructed by cloning the wild-type (WT) human *PCSK9* 3′-UTR (RefSeq ID: NM_174,936) or a human *PCSK9* 3′-UTR harboring mutations in the predicted miR-99a-5p binding site in front of the *luc2* reporter gene in the pmirGLO vector (Figure 4B), and luciferase reporter assay was performed. As seen in Figures 4C, D, the luciferase activity was vastly reduced by miR-99a-5p as compared to Con miR and markedly upregulated by anti-miR-99a-5p in comparison with Con Inh in HepG2 cells transfected with the luciferase reporter plasmids containing WT 3′-UTR of human *PCSK9*.

In accordance with our hypothesis, the regulation of luciferase activity by both miR-99a-5p and anti-miR-99a-5p was eliminated in HepG2 cells harboring luciferase reporter plasmids containing the mutated binding site (Figures 4C, D). Hence, it was demonstrated that miR-99a-5p negatively regulates human PCSK9 expression by interacting with the predicted target site “UACGGGU” in the human *PCSK9* 3′-UTR.

### 3.4 MiR-99a-5p inhibits PCSK9-mediated LDLR degradation in human hepatic cells

**FIGURE 5:**
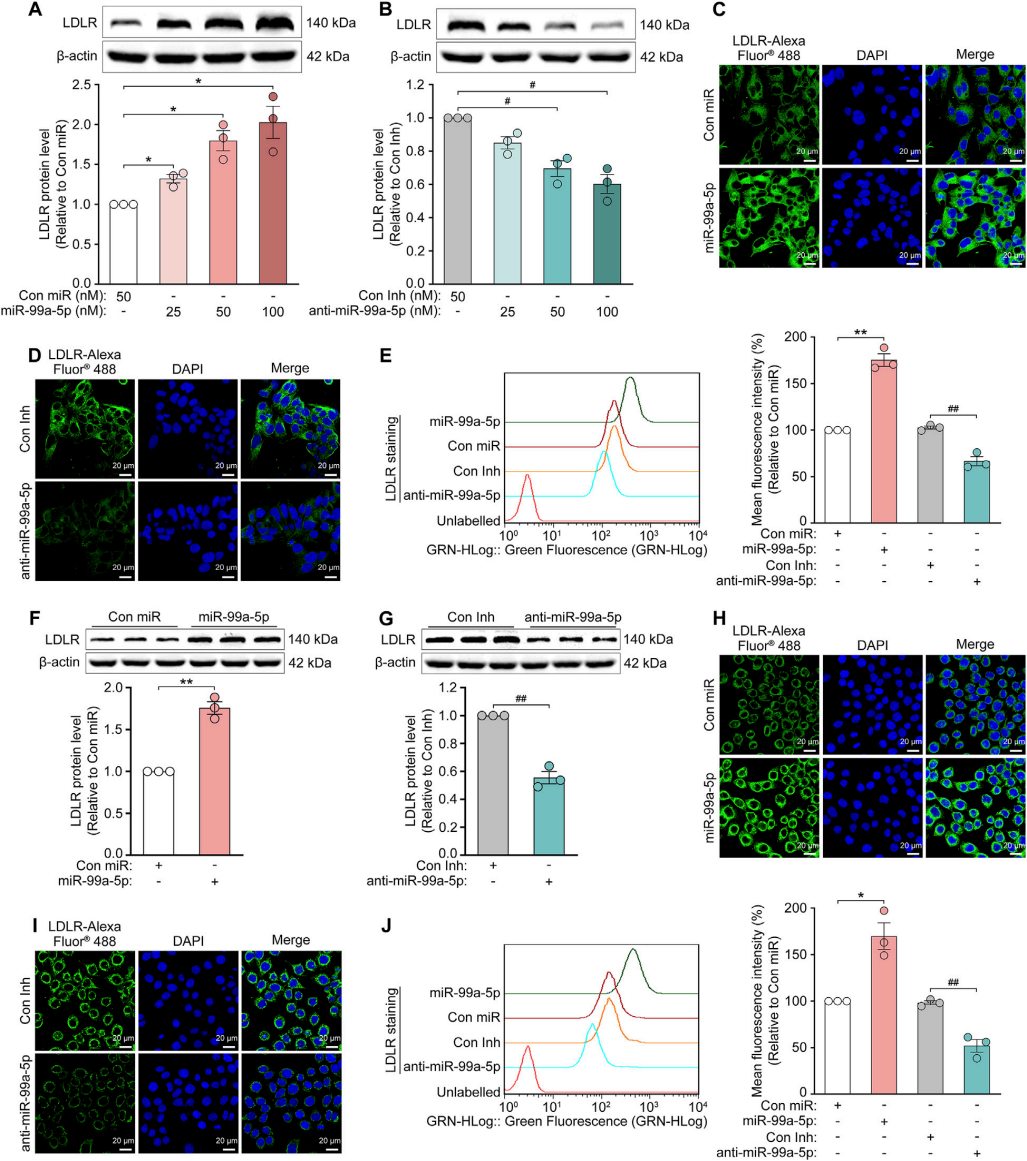
MiR-99a-5p increases LDLR levels in human hepatocytes. **(A, B)** Total LDLR levels were detected by Western blot in HepG2 cells. **(C–E)** After the cells were transfected with 50 nM Con miR, miR-99a-5p, Con Inh or anti-miR-99a-5p for 72 h, then the cell-surface LDLR levels were assessed via immunofluorescence analysis **(C, D)** and flow cytometry analysis **(E)** in HepG2 cells. **(F–J)** LO2 cells were transfected with 50 nM Con miR, miR-99a-5p, Con Inh or anti-miR-99a-5p for 72 h **(F, G)** Total LDLR levels were detected via Western blot. Cell-surface LDLR levels were determined by immunofluorescence **(H, I)** and flow cytometry **(J)** analyses as described above. Data were presented as the means ± SEM in three independent experiments. ^*^ *p* < 0.05, ^**^ *p* < 0.01 vs. Con miR; ^#^ *p* < 0.05, ^##^ *p* < 0.01 vs. Con Inh. Significance was performed by one-way ANOVA and two-tailed Student’s *t*-test.

PCSK9 in plasma binds to LDLR on the surface of hepatocytes, preventing its recycling and stimulating its degradation in lysosomes (Seidah et al., 2014). Thus, we measured the impact of miR-99a-5p on LDLR protein levels via Western blot. As expected, it was shown that LDLR protein levels were dose-dependently upregulated after transfection with indicated concentrations of miR-99a-5p (25, 50, 100 nM) for 72 h (Figure 5A), and downregulated in a dose-dependent manner following transfection with various concentrations of anti-miR-99a-5p (25, 50, 100 nM) for 72 h in HepG2 cells (Figure 5B). In addition, miR-99a-5p increased LDLR protein level by 75.83% in comparison with Con miR (Figure 5F), whereas anti-miR-99a-5p decreased LDLR protein level by 44.47% compared with Con Inh (Figure 5G) in LO2 cells.

Additionally, we tested the cell-surface LDLR protein levels following transfection with Con miR, miR-99a-5p, Con Inh or anti-miR-99a-5p at 50 nM for 72 h, via immunofluorescence and flow cytometry analyses, respectively. The results showed that overexpression of miR-99a-5p substantially increased LDLR protein level at the cell surface as compared with Con miR (Figures 5C, E), whereas the downregulation of endogenous miR-99a-5p by anti-miR-99a-5p strongly reduced the cell-surface LDLR protein level relative to Con Inh in HepG2 cells (Figures 5D, E). Additionally, we observed the similar results in LO2 cells (Figures 5H–J). Accordingly, it is confirmed that miR-99a-5p inhibits PCSK9-mediated LDLR degradation in human liver cells.

### 3.5 MiR-99a-5p augments LDL-C uptake and intracellular cholesterol levels of human hepatocytes

**FIGURE 6:**
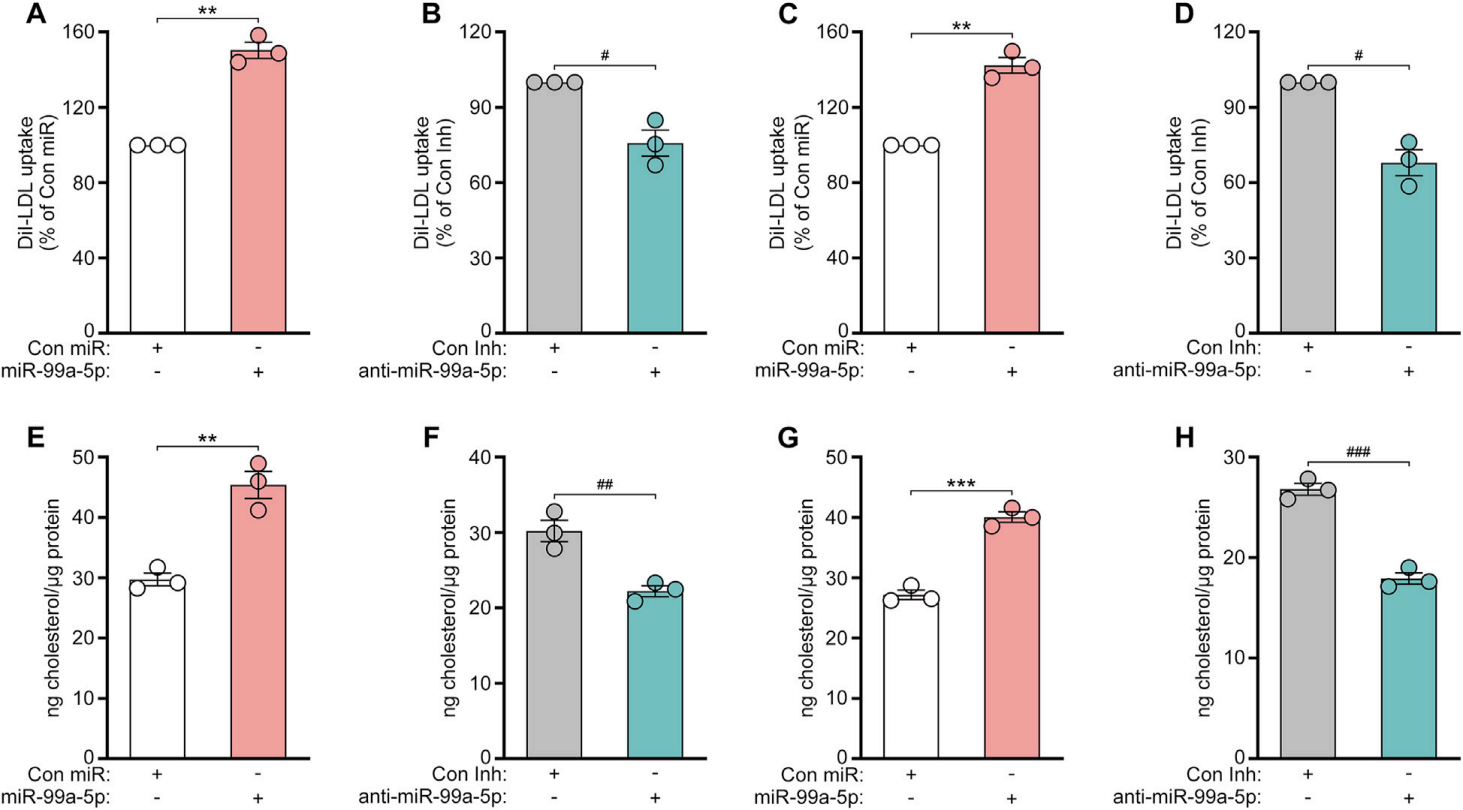
MiR-99a-5p enhances LDL-C uptake and intracellular cholesterol levels in human hepatocytes. **(A, B)** 50 nM Con miR or miR-99a-5p **(A)**, or 50 nM Con Inh or anti-miR-99a-5p **(B)** were transfected in HepG2 cells. Subsequently, DiI-LDL (20 μg/mL) was incubated. DiI-LDL uptake was measured. **(C, D)** DiI-LDL uptake was assessed in LO2 cells. **(E, F)** HepG2 cells were transfected and then incubated with ox-LDL (50 μg/mL). Thereafter, intracellular cholesterol content was examined as described above. **(G, H)** Intracellular cholesterol levels in LO2 cells were detected. Results were given as the means ± SEM and representative in three independent experiments. ^**^ *p* < 0.01, ^***^ *p* < 0.001 vs. Con miR; ^#^ *p* < 0.05, ^##^ *p* < 0.01, ^###^ *p* < 0.001 vs. Con Inh. Significance was analyzed by two-tailed Student’s *t*-test.

To further investigate the functional consequence of miR-99a-5p targeting *PCSK9*, we first examined the effects of miR-99a-5p overexpression or inhibition on DiI-LDL uptake in human hepatocytes. As a result, DiI-LDL uptake was enhanced by 50.37% in HepG2 cells transfected with 50 nM miR-99a-5p in comparison with Con miR (Figure 6A). Conversely, DiI-LDL uptake was decreased by 24.22% in HepG2 cells transfected with 50 nM anti-miR-99a-5p relative to Con Inh (Figure 6B), which is consistent with their impacts on LDLR protein levels. Additionally, we observed the similar effects in LO2 cells (Figures 6C, D).

Subsequently, we investigated whether miR-99a-5p affects the intracellular cholesterol levels of human hepatic cells. HepG2 and LO2 cells were both transfected with 50 nM Con miR, miR-99a-5p, Con Inh, anti-miR-99a-5p, respectively. After 48 h, the cells were incubated with ox-LDL (50 μg/mL) for another 24 h. Then, the content of intracellular cholesterol was measured as described above. As shown in Figure 6E, miR-99a-5p increased the intracellular cholesterol concentration by 52.76% as compared to Con miR in HepG2 cells. Instead, anti-miR-99a-5p attenuated the intracellular cholesterol levels by 26.42% compared with Con Inh in HepG2 cells (Figure 6F). We observed the similar results in LO2 cells (Figures 6G, H). These results suggest that miR-99a-5p functionally enhances LDL-C uptake and intracellular cholesterol levels in human hepatic cells.

## 4 Discussion

**FIGURE 7:**
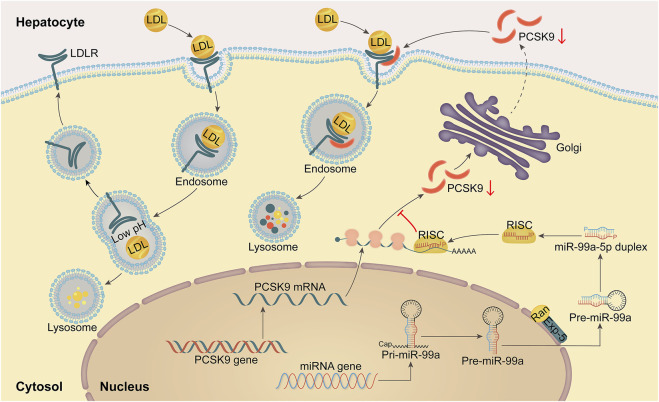
A schematic diagram of the mechanism by which miR-99a-5p inhibits PSCK9 expression by directly interacting with the putative target site in human *PCSK9* 3′-UTR, thereby up-regulating LDLR levels and functionally enhancing LDL-C uptake in human hepatocytes.

This work revealed that miR-99a-5p functions as a negative regulator to suppress PCSK9 expression by binding to a putative target site in human *PCSK9* 3′-UTR, thereby elevating LDLR levels and functionally enhancing LDL-C uptake in human liver cells (Figure 7).

PCSK9, the 9th member of the mammalian proprotein convertase family, is largely synthesized in the liver and plays a pivotal regulatory role in cholesterol homeostasis (Seidah et al., 2003; Seidah et al., 2014). After being secreted into the plasma, PCSK9 enhances the degradation of LDLR in lysosomes by binding to its extracellular EGF-A structural domain, decreasing the elimination of circulating LDL-C (Seidah et al., 2014; Bergeron et al., 2015). Previously, it was reported that gain-of-function mutations in *PCSK9* resulted in autosomal dominant familial hypercholesterolemia (Abifadel et al., 2003), whereas loss-of-function mutations in *PCSK9* reduced plasma LDL-C levels and decreased risk of cardiovascular disease (CVD) (Jonathan et al., 2005).

In addition, PCSK9 expression is regulated by different mechanisms at the epigenetic, transcriptional and posttranscriptional levels. Epigenetically, FoxO3 recruits Sirt6 deacetylase to *Pcsk9* gene proximal promoter region and deacetylates histone H3, thus repressing PCSK9 expression (Tao et al., 2013). As a cofactor of HINFP (histone nuclear factor P), NPAT recruits the cofactor of HAT (histone acetyltransferase), TRRAP, to promote histone H4 acetylation at the *PCSK9* promoter, thereby coactivating SREBP2 (sterol-regulatory element binding protein 2) mediated transactivation of *PCSK9* gene expression (Li and Liu, 2012). Furthermore, *PCSK9* is positively associated with promoter methylation, which is conserved across tissues (Lohoff et al., 2018).

Although PCSK9 is activated by SREBP-1/2 via SRE in its proximal promoter region, SREBP-2 predominantly mediates its sterol-dependent regulation *in vivo* (Jeong et al., 2008). In addition, *PCSK9* gene can be transcriptionally enhanced by HNF-1α via binding to a highly conserved site located 28 bp upstream of the *PCSK9* promoter SRE-1 site (Li et al., 2009). Notably, the binding site of HNF-1α in the *PCSK9* promoter contains a consensus site for FoxO transcription factor binding. FoxO binding here is presumed to reduce HNF-1α transactivating activity on the *Pcsk9* promoter. (Tao et al., 2013). Additionally, farnesoid X receptor activation downregulates PCSK9 expression (Langhi et al., 2008), and the expression of both PCSK9 and LDLR can be induced through ligands and dephosphorylation by peroxisome proliferator-activated receptor γ (Duan et al., 2012).

Post-transcriptionally, miRNAs can regulate PCSK9 expression. To date, miR-224, miR-222, miR-191 (Naeli et al., 2017), miR-564, miR-4721 (Los et al., 2021), miR-520d (Salerno et al., 2020), miR-3165, miR-221-5p, miR-363-5p, miR-765, miR-342-5p, miR-609 (van Solingen et al., 2021), miR-143-5p, miR-1228-3p (Decourt et al., 2020) have been found to negatively regulate PCSK9 in HepG2, Huh7 cells. However, whether these miRNAs can functionally promote LDL-C uptake in hepatocytes has not been investigated (Salerno et al., 2020). Moreover, miR-99a-5p as another new PCSK9 inhibitor had stronger inhibitory effect on the expression of luciferase activity than miR-224-5p and miR-191-5p (Supplementary Figure S2). Although miR-337-3p (Xu X. et al., 2021), miR-483 (Dong et al., 2020) and miR-552-3p (Ma et al., 2021) have been identified to reduce serum LDL-C level in mice by targeting PCSK9, miR-483 and miR-552-3p have been found to stimulate cell proliferation, migration and invasion of cancer (Song et al., 2014; Huang et al., 2021).

Herein, our study revealed miR-99a-5p as a novel post-transcriptional inhibitor of the human *PCSK9* gene, which directly binds to the predicted interacting site “UACGGGU” in the human *PCSK9* 3′-UTR to suppress PCSK9 expression, thus reducing LDLR degradation mediated by PCSK9 and functionally promoting LDL-C uptake in human hepatocytes. By bioinformatics analysis, it was uncovered that human *PCSK9* 3′-UTR contains one binding site for miR-99a-5p that is conserved in human and chimpanzee but not in mouse, which may explain why mouse *Pcsk9* gene expression is not regulated by miR-99a-5p in mouse hepatic cell line, Hepa1-6.

MiR-99a is a member of the miR-99 family, an evolutionarily conserved family that includes miR-99a, miR-99b, and miR-100 (Eniafe and Jiang, 2021). MiR-99a (Gene ID: 407055; Ensembl ID: ENSG00000207638; miRBase Accession: MI0000101), located on chromosome 21q21.1, is an intronic miRNA that is encoded within an intron of LINC00478 (also known as C21orf34, MIR99AHG and MONC; human Ensembl ID: ENSG00000215386, mouse Ensembl ID: ENSMUSG00000090386) (Li et al., 2011; Oneyama et al., 2011; Sun et al., 2014), and produces two mature miRNAs, miR-99a-5p and miR-99a-3p. Intriguingly, it was reported that miR-99a-5p overexpression enhances sensitivity to cisplatin (DDP) and cell apoptosis by suppressing VLDLR expression in lung cancer cells (Lang et al., 2023), and miR-99a inhibits two novel oncogenic proteins E2F2 and EMR2 and represses stemness in lung cancer (Feliciano et al., 2017). In addition, miR-99a-5p attenuates atherosclerosis via targeting Homeobox A1 (HOXA1) (Han et al., 2019). MiR-99a overexpression attenuates cardiac hypertrophy (Li et al., 2016), suppresses endothelial cell inflammation induced by lipopolysaccharide (LPS) via inhibition of the mTOR/NF-κB signaling pathway (Bao et al., 2016), inhibits M1 macrophage phenotype activation by targeting TNFα, and mitigates adipose tissue inflammation and improves insulin sensitivity as well as diabetes-associated dyslipidemia in diabetic mice (Jaiswal et al., 2019), indicating dysregulation of miR-99a-5p is closely associated with dyslipidemia and dyslipidemia-relevant diseases such as inflammation, atherosclerosis.

## 5 Conclusion

Together, our findings indicate that miR-99a-5p is a negative regulator of PSCK9, and reveal a molecular mechanism by which miR-99a-5p restoration upregulates LDLR and functionally enhances LDL-C uptake via post-transcriptional repression of *PCSK9* expression in human hepatocytes. We propose that miR-99a-5p can potentially serve as an inhibitor of PCSK9 to promote LDL-C uptake in hepatocytes to ameliorate hypercholesterolemia and atherosclerosis.

## Data Availability

The original contributions presented in the study are included in the article/Supplementary Material, further inquiries can be directed to the corresponding author.
