# Supplementary material for: MiR-99a-5p up-regulates LDLR and functionally enhances LDL-C uptake via suppressing PCSK9 expression in human hepatocytes
Source: Front Genet. 2024 Nov 19;15:1469094. doi: 10.3389/fgene.2024.1469094 (PMC11611869; doi:10.3389/fgene.2024.1469094)
Supplement: Supplementary file 1 [file DataSheet2.pdf]

Supplementary Material

Supplementary Figures

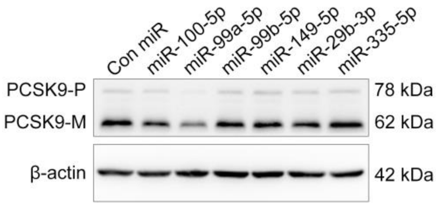

Supplementary Figure 1. The regulatory effects of these miRNAs that were selected with good prediction scores using bioinformatics analyses on PCSK9 protein expression in HepG2 cells were assessed by western blot. HepG2 cells were transfected with 50 nM control miRNA mimic (Con miR) or the selected miRNA mimic (miR-100-5p, miR-99a-5p, miR-99b-5p, miR-149-5p, miR-29b-3p, miR-335-5p) for 72 h. The inhibitory effects of the 6 miRNAs on PCSK9 protein levels were measured by western blot.

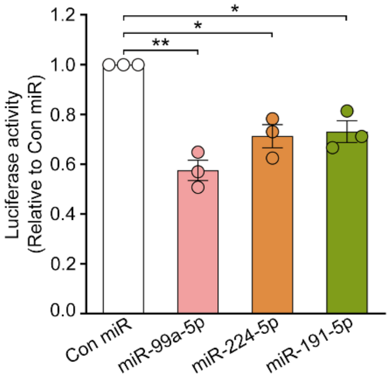

Supplementary Figure 2. Luciferase activity was detected in HepG2 cells transfected with luciferase reporter plasmids containing wild-type human *PCSK9* mRNA 3'-UTR and 50 nM Con miR, miR-99a-5p, miR-224-5p or miR-191-5p. Data were given as the means ± SEM and representative in three independent experiments. $^{*}p < 0.05$, $^{**}p < 0.01$ *vs*. Con miR. Significance was analyzed by one-way ANOVA.
