# Supplementary material for: MiR-99a-5p up-regulates LDLR and functionally enhances LDL-C uptake via suppressing PCSK9 expression in human hepatocytes
Source: Front Genet. 2024 Nov 19;15:1469094. doi: 10.3389/fgene.2024.1469094 (PMC11611869; doi:10.3389/fgene.2024.1469094)
Supplement: Supplementary file 3 [file Table1.docx]

Supplementary Material

# Supplementary Tables

**Supplementary Table 1.** Primers used for qRT-PCR.

| **Gene** | **Forward primer sequence (5’-3’)** | **Reverse primer sequence (5’-3’)** |
| --- | --- | --- |
| Human *PCSK9* | AGGGGAGGACATCATTGGTG | CAGGTTGGGGGTCAGTACC |
| Human *β-actin* | CTCTTCCAGCCTTCCTTCCT | CAGGGCAGTGATCTCCTTCT |
