# Supplementary figures and images for: MiR-99a-5p up-regulates LDLR and functionally enhances LDL-C uptake via suppressing PCSK9 expression in human hepatocytes
Source: Front Genet. 2024 Nov 19;15:1469094. doi: 10.3389/fgene.2024.1469094 (PMC11611869; doi:10.3389/fgene.2024.1469094)

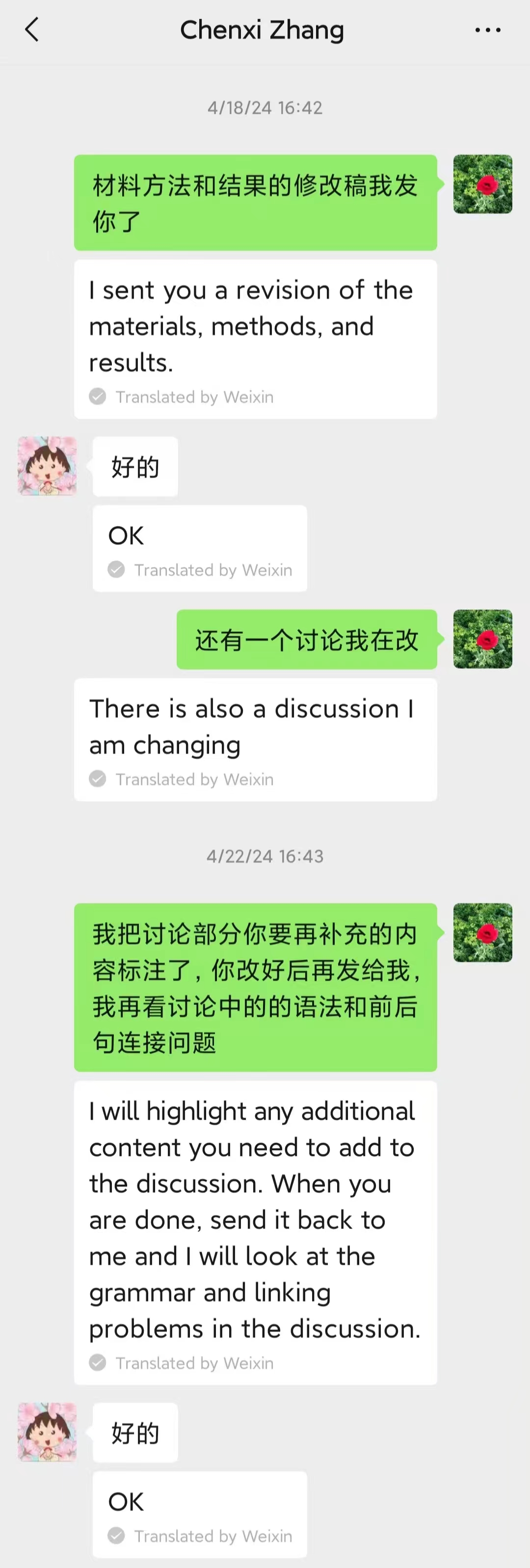

Supplement: Supplementary file 4 [file DataSheet1.zip › WeChat application messages between the authors that predate initial submission/WeChat application messages between Xuemei Chen and Chenxi Zhang that predate initial submission.jpg]

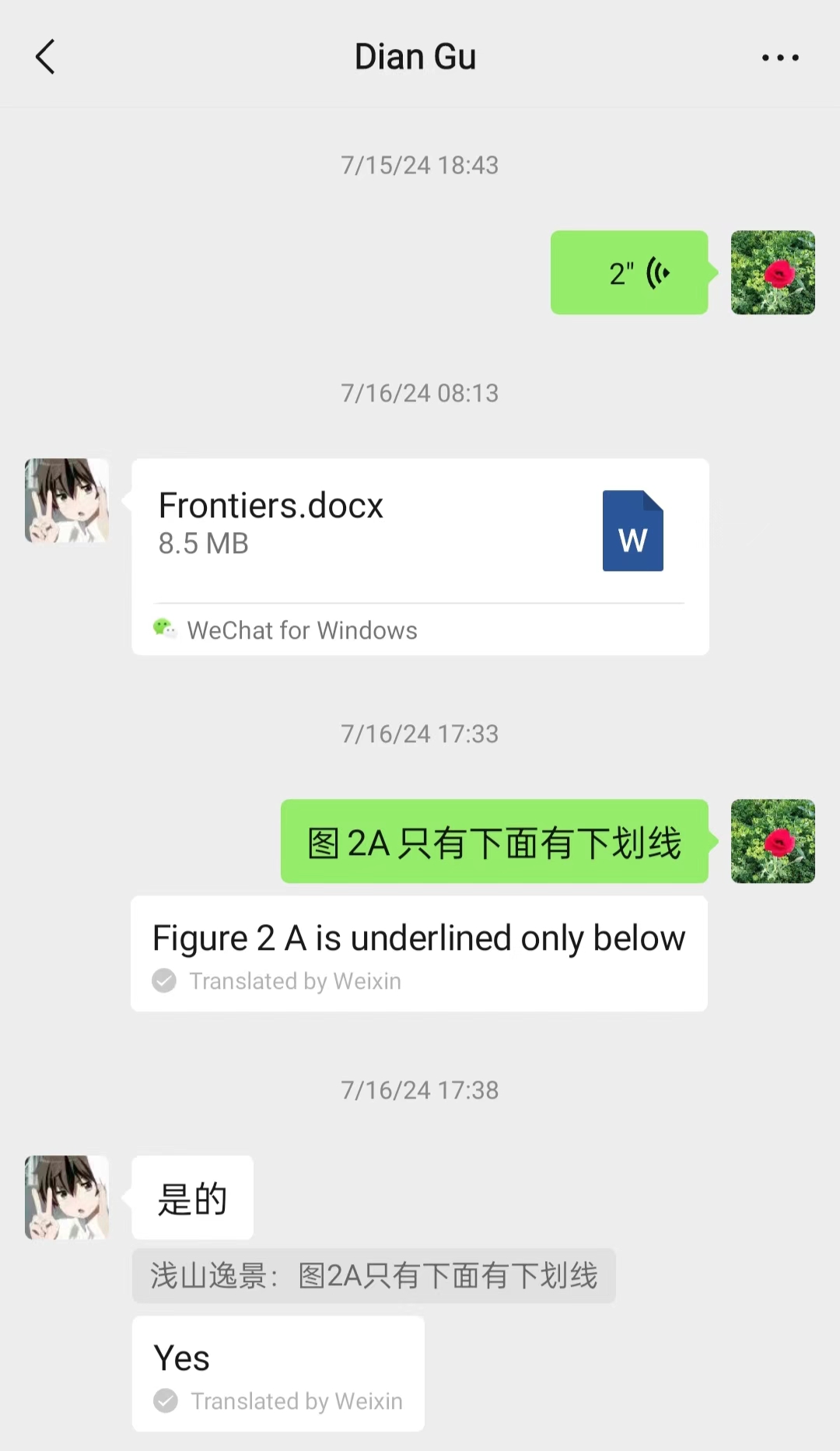

Supplement: Supplementary file 4 [file DataSheet1.zip › WeChat application messages between the authors that predate initial submission/WeChat application messages between Xuemei Chen and Dian Gu that predate initial submission.jpg]

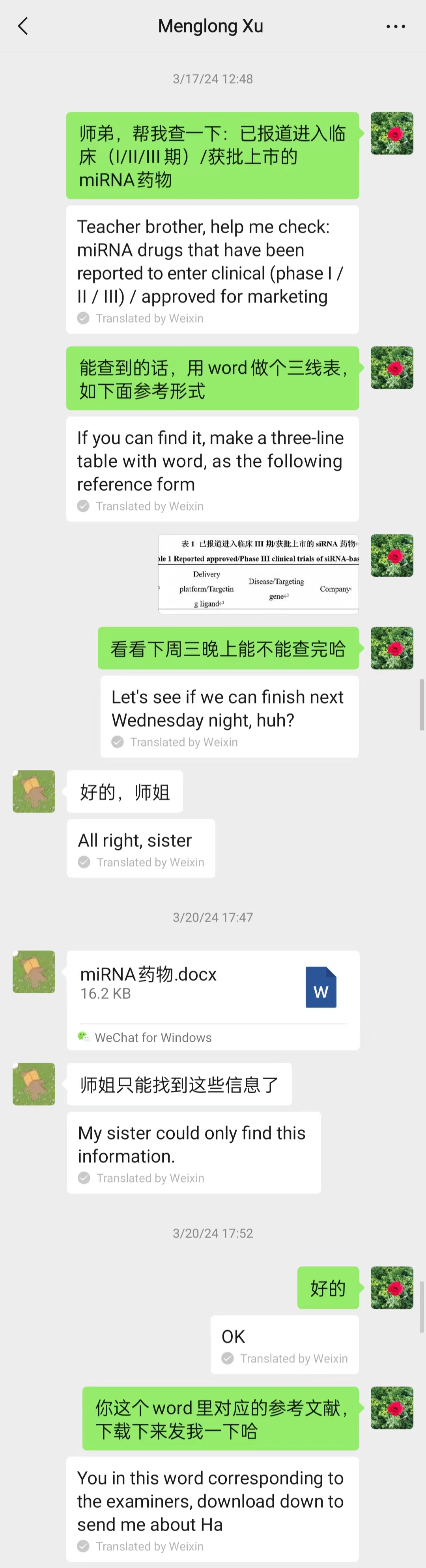

Supplement: Supplementary file 4 [file DataSheet1.zip › WeChat application messages between the authors that predate initial submission/WeChat application messages between Xuemei Chen and Menglong Xu that predate initial submission.jpg]

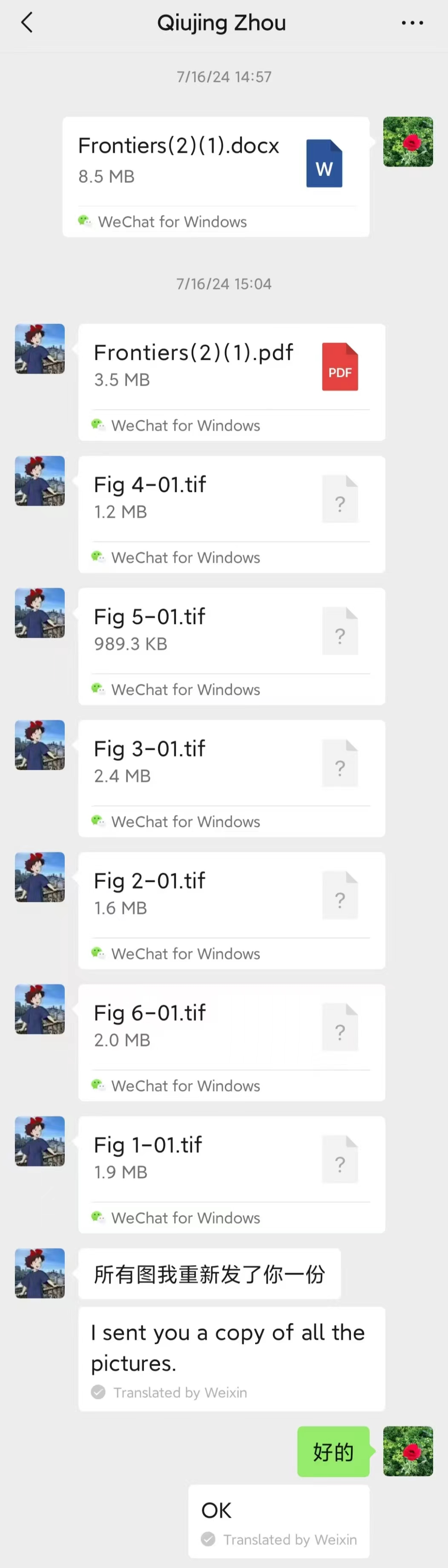

Supplement: Supplementary file 4 [file DataSheet1.zip › WeChat application messages between the authors that predate initial submission/WeChat application messages between Xuemei Chen and Qiujing Zhou that predate initial submission.jpg]

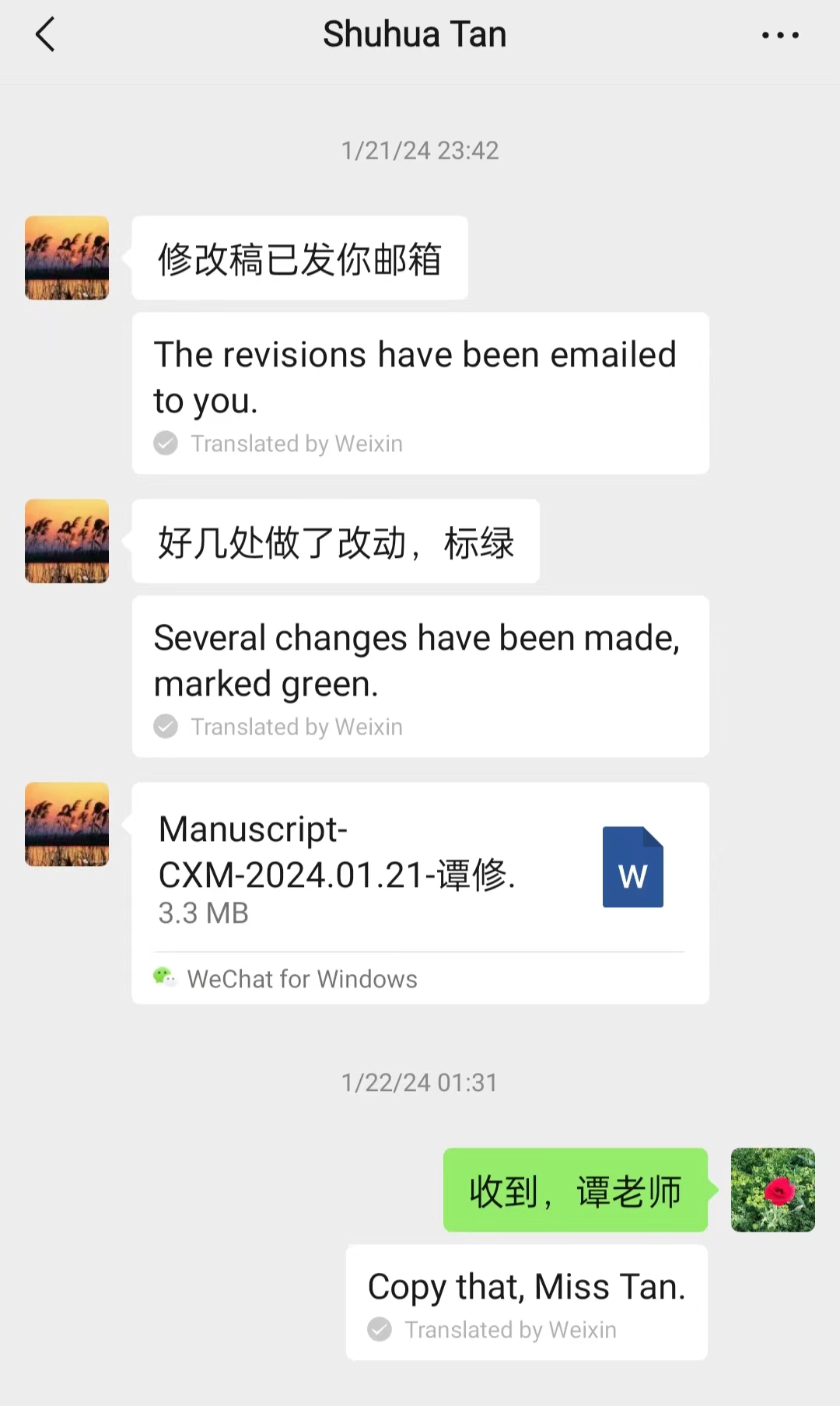

Supplement: Supplementary file 4 [file DataSheet1.zip › WeChat application messages between the authors that predate initial submission/WeChat application messages between Xuemei Chen and Shuhua Tan that predate initial submission.jpg]

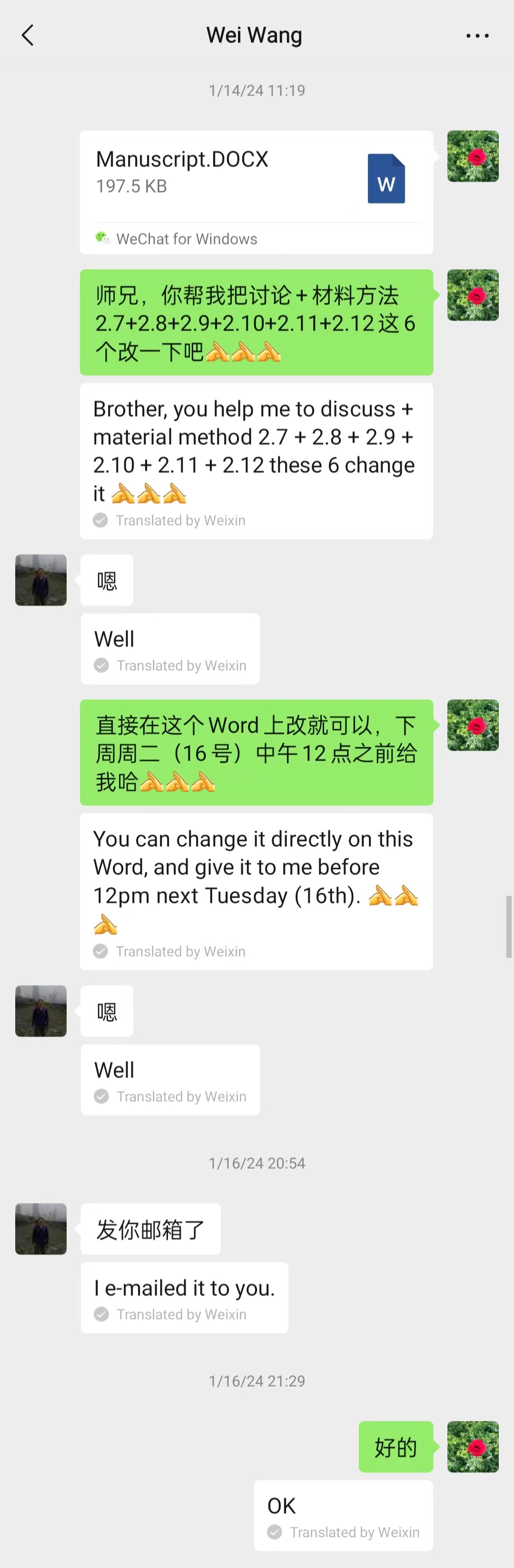

Supplement: Supplementary file 4 [file DataSheet1.zip › WeChat application messages between the authors that predate initial submission/WeChat application messages between Xuemei Chen and Wei Wang that predate initial submission.jpg]

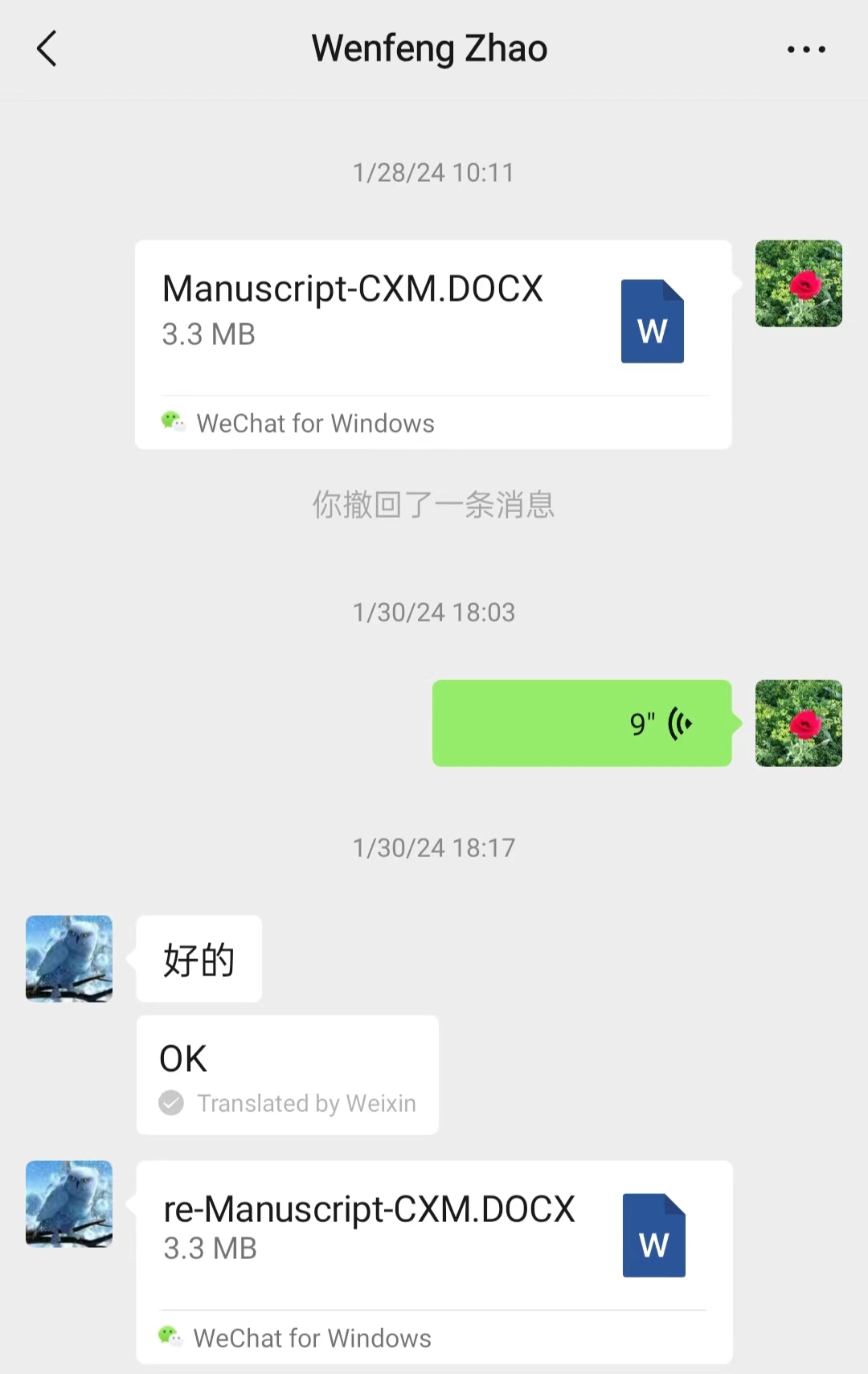

Supplement: Supplementary file 4 [file DataSheet1.zip › WeChat application messages between the authors that predate initial submission/WeChat application messages between Xuemei Chen and Wenfeng Zhao that predate initial submission.jpg]

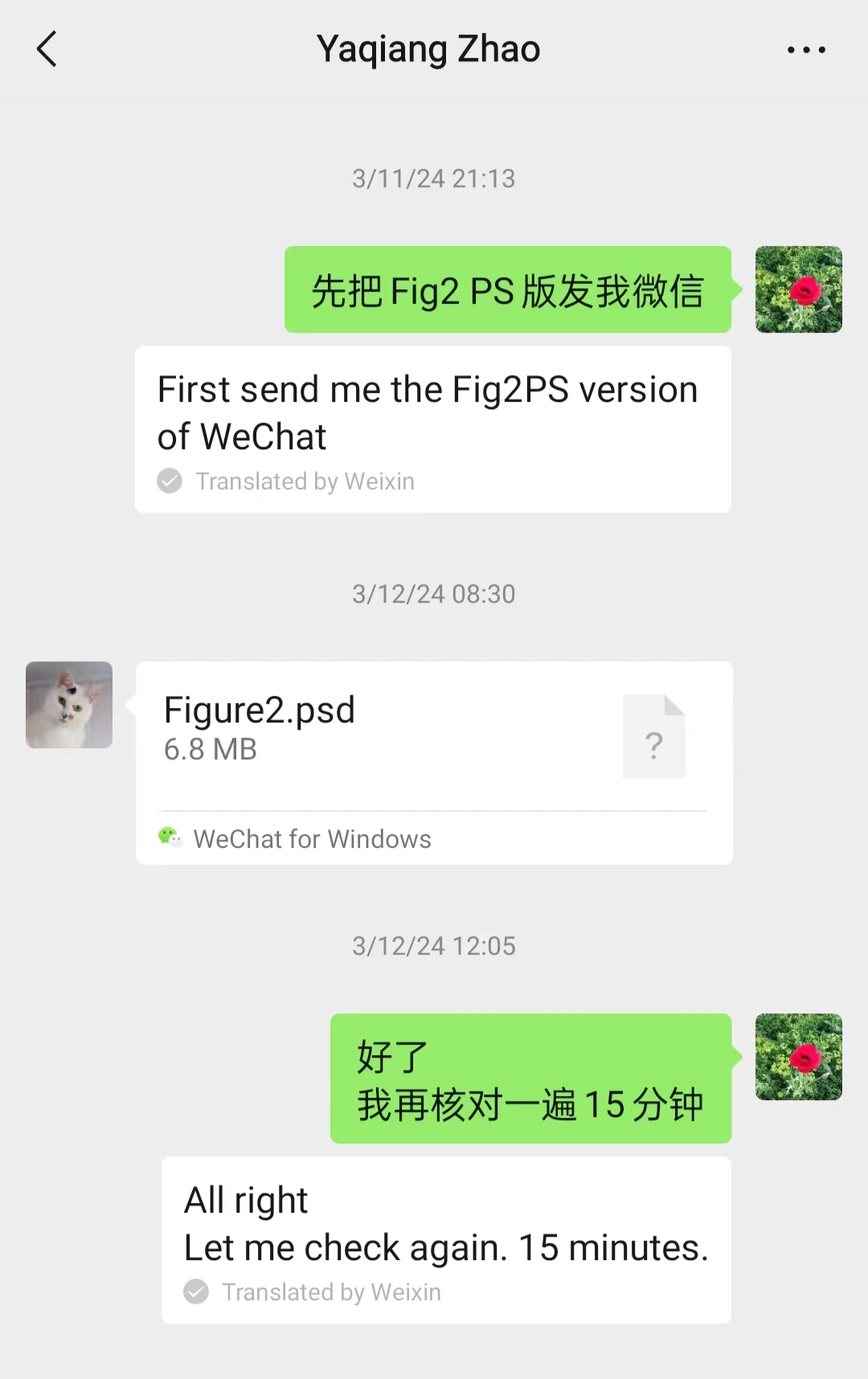

Supplement: Supplementary file 4 [file DataSheet1.zip › WeChat application messages between the authors that predate initial submission/WeChat application messages between Xuemei Chen and Yaqiang Zhao that predate initial submission.jpg]

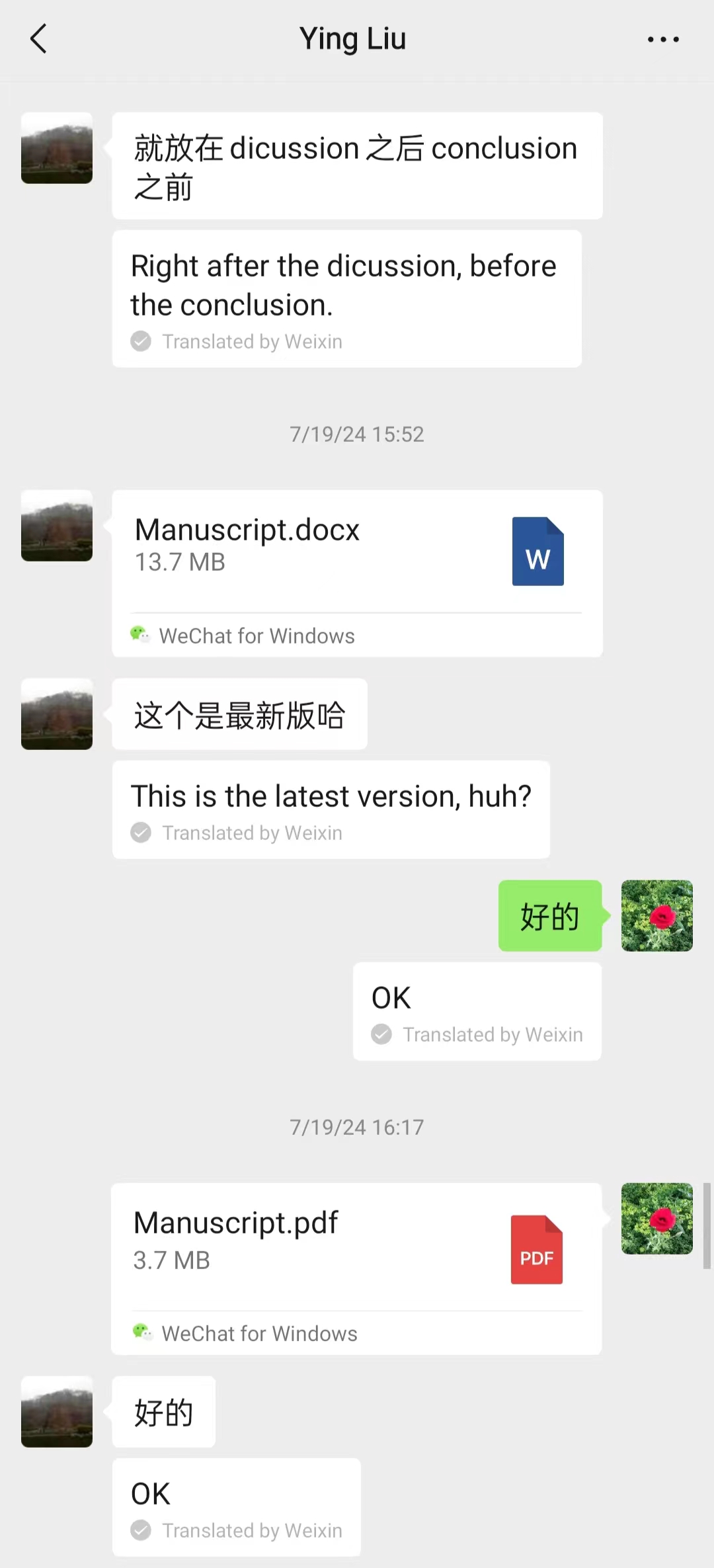

Supplement: Supplementary file 4 [file DataSheet1.zip › WeChat application messages between the authors that predate initial submission/WeChat application messages between Xuemei Chen and Ying Liu that predate initial submission.jpg]
