# Supplementary material for: MiR-99a-5p up-regulates LDLR and functionally enhances LDL-C uptake via suppressing PCSK9 expression in human hepatocytes
Source: Front Genet. 2024 Nov 19;15:1469094. doi: 10.3389/fgene.2024.1469094 (PMC11611869; doi:10.3389/fgene.2024.1469094)
Supplement: Supplementary file 6 [file Table2.docx]

Supplementary Material

# Supplementary Tables

**Supplementary Table 2.** Primers used to amplify the 3’-UTR fragments of human *PCSK9* gene.

| **Primer** | **Sequence (5’-3’)** | **Description** |
| --- | --- | --- |
| PCSK9-WT-F | CCGCTCGAGGCTGAGCTTTAAAATGGTTC | F for WT, Mut-1 |
| PCSK9-WT-R | CTAGTCTAGAGGCAACAGAGAGGACAGACC | R for WT, Mut-2 |
| PCSK9-Mut-OR | CCCAGCCTCACTGTTTGGGCATAAAATGAGGAGCCCA | R for Mut-1 |
| PCSK9-Mut-OF | TGGGCTCCTCATTTTATGCCCAAACAGTGAGGCTGGG | F for Mut-2 |

F, forward primer; R, reverse primer; WT, wild-type *PCSK9* 3’-UTR (1098 bp); Mut, *PCSK9* 3’-UTR with mutant miR-99a-5p binding sites; Mut-1, 5’ upstream segment in overlap extension PCR for mutated *PCSK9* 3’-UTR fragment; Mut-2, 3’ downstream segment in overlap extension PCR for mutated *PCSK9* 3’-UTR fragment.
