# Supplementary material for: MiR-99a-5p up-regulates LDLR and functionally enhances LDL-C uptake via suppressing PCSK9 expression in human hepatocytes
Source: Front Genet. 2024 Nov 19;15:1469094. doi: 10.3389/fgene.2024.1469094 (PMC11611869; doi:10.3389/fgene.2024.1469094)
Supplement: Supplementary file 7 [file Table3.docx]

Supplementary Material

# Supplementary Tables

**Supplementary Table 3.** Human PCSK9-targeting miRNAs predicted by bioinformatics analyses and the regulatory effects of these miRNAs on PCSK9 protein expression in HepG2 cells.

| **miRNA** | **miRBase**  **Accession** | **Predicted target sites** | | **miRanda** | **TargetScan** | **miRDB** | **Relative PCSK9 protein level**  **(mimic)** |
| --- | --- | --- | --- | --- | --- | --- | --- |
|  |  | **TargetScan** | **miRanda** | **mirSVR score** | **Context++ score percentile** | **Target score** |  |
| miR-100-5p | MIMAT0000098 | 1 | 1 | -0.3543 | 98 | 85 | 0.7603±0.07632 |
| miR-99a-5p | MIMAT0000097 | 1 | 1 | -0.3543 | 98 | 85 | 0.5113±0.03927^**^ |
| miR-99b-5p | MIMAT0000689 | 1 | 1 | -0.3543 | 98 | 85 | 0.8517±0.0407 |
| miR-149-5p | MIMAT0000450 | 4 | 2 | -0.0017/  -0.4409 | 37/96/84/37 | 62 | 1.0240±0.03329 |
| miR-29b-3p | MIMAT0000100 | 2 | 3 | -0.0006/  -0.0053/  -1.2529 | 72/98 | - | 0.9950±0.01931 |
| miR-335-5p | MIMAT0000765 | 1 | 1 | -0.3549 | 94 | - | 1.0880±0.06791 |

Following transfection of HepG2 cells with 50 nM each of the selected miRNA mimics for 72 h, PCSK9 protein levels were assessed by western blot. Values for cells transfected with control miRNA mimic (Con miR) were set to 1. Results were given as the means ± SEM of three independent experiments. ^**^*p* < 0.01 *vs*. Con miR.
